# The impact of education level and economic freedom on gender inequality: panel evidence from emerging markets

**DOI:** 10.3389/fpsyg.2023.1202014

**Published:** 2023-08-02

**Authors:** Pinar Tokal, Gamze Sart, Marina Danilina, Mohammad A. Ta’Amnha

**Affiliations:** ^1^Institute of Social Sciences, Netkent Mediterranean Research and Science University, Nicosia, Cyprus; ^2^Hasan Ali Yucel Faculty of Education, Department of Educational Sciences, İstanbul University-Cerrahpaşa, İstanbul, Turkey; ^3^Department of Economics, Plekhanov Russian University of Economics, Moscow, Russia; ^4^Department of Economics, Financial University under the Government of the Russian Federation, Moscow, Russia; ^5^Management Sciences Department, Business School, German Jordanian University, Amman, Jordan

**Keywords:** education level, economic freedom, gender inequality, panel causality analysis, panel cointegration analysis, emerging market economies

## Abstract

Gender equality is a critical factor for all ingredients of a healthy society and sustainable development. Therefore, measures to decrease gender inequalities in economic, social, and political life are important for the economic and social development of a society. This study analyzes the influence of education level and economic freedom on gender inequality in emerging markets over the 2000–2020 term through causality and cointegration tests. The results of the causality test uncover a bidirectional causality between education level, economic freedom, and gender inequality. In other words, there exists a mutual interaction among education level, economic freedom, and gender inequality in the short term. Furthermore, the findings of cointegration analysis indicate that education level and economic freedom have a negative impact on gender inequality in the long term, but education level is much more effective on gender inequality than economic freedom in nearly all emerging markets.

## 1. Introduction

The women population constitutes nearly half of the world population (World Bank, 2023). Therefore, the women population also represents half of human capital, a significant determinant of economic sustainability and social development. However, men and women do not generally have equal rights and opportunities in education, health, economic and social life in many countries, but gender inequality remarkably differs among countries depending on their cultural norms, and social and economic development levels. In this context, the gender inequality index (GII) (lower GII values show lower gender inequality) of 2021 calculated by UNDP (2023) indicated that Denmark (GII: 0.013), Norway (GII: 0.016), Switzerland (GII: 0.018), Sweden (GII: 0.023), and the Netherlands (GII: 0.025) were the first five countries with the lowest gender inequality, but Yemen (GII: 0.820), Papua New Guinea (GII: 0.725), Nigeria (GII: 0.680), Afghanistan (GII: 0.678), and Central African Republic (GII: 0.672) were the last five countries with the highest gender inequality. Countries with lower gender inequality generally have lower human development when GII figures and human development levels of the countries are together considered.

Women generally have less access to economic and social resources, the labor market, and the political world and use their resources in non-economic activities such as housework and care, education, and the health of their children (Schultz, 2002; Ferrant, 2015; Bertay et al., 2020). Thus, gender inequalities in education, health, social, economic, and political activities have the potential to negatively influence capital accumulation, technological development, productivity, and institutional development which are the main factors underlying economic growth and development (Ferrant, 2015). Thereby, gender inequality becomes more of an issue for economic growth and development.

In this context, Wu et al. (2022) discovered a negative interaction between gender inequality and economic growth in China in the short and long term. Koengkan et al. (2022) also uncovered a negative influence of gender inequality on economic growth in Latin America and the Caribbean states. Many other researchers such as Kleven and Landais (2017), Karoui and Feki (2018), Bertay et al. (2020), and Farooq et al. (2020) have discovered a negative influence of gender inequality on economic growth in different countries and country groups. Ferrant (2015) also suggested gender inequality is a significant determinant of economic and human development and found that gender inequality decreased human development by 4.6% in 109 countries mainly driven by gender inequalities in family, education and access to economic activities. Therefore, gender inequality can negatively influence social development through education and health channels. Furthermore, gender gaps in wages, labor participation, and the informal sector can positively influence income inequality (Gonzales et al., 2015).

Moreover, gender equality is one of the 17 sustainable development goals (SDGs) accepted by all United Nations Member States in 2015 (United Nations, 2023a) and the United Nations suggest that gender equality is a crucial factor for all aspects of a healthy society, including decreasing poverty, increasing education and health protection, and the well-being of all persons (United Nations, 2023b). Hence, the revelation of the factors behind gender inequality would be useful to arrange the right policies to decrease gender inequality. However, researchers have generally analyzed the relationship between gender inequality and economic growth in the empirical literature, and the determinants of gender inequality have been investigated by few researchers. The studies on the determinants of gender inequality have set forth that GDP *per capita*, education, fertility rate, cultural norms, economic freedom, public governance, trade, globalization, and foreign direct investments are significant factors underlying gender inequality (Baliamoune-Lutz, 2007; Juhn et al., 2014; Jayachandran, 2015; Sangaji et al., 2018; Adeosun and Owolabi, 2021; Kim, 2021; Apergis and Lynch, 2022).

In this research, we focus on the short-and long-term influence of economic freedom and education level on gender inequality, because both variables have the potential to affect gender inequality through multiple diverse channels. First, education is one of the dominant factors behind human capital which is a key determinant of economic growth and development. Therefore, education raises the awareness of society about the role of women in economic and social development (Kane, 1995). Furthermore, decreases in gender inequality also can foster education by increasing the roles of women in social and economic life. As a result, a bilateral interaction between education and gender inequality seems possible in theoretical terms. Furthermore, economic freedom can also decrease gender inequality, because market-oriented economies generally introduce equal opportunities for everybody regardless of an individual’s gender (Stroup, 2008). The improvements in gender equality can also foster economic freedom by raising the roles of women in economic, social, and political life. Similarly, a mutual interplay between economic freedom and gender inequality is also expected.

This article explores the influence of education level and economic freedom on gender inequality in a sample of 21 emerging markets, presented in Table 1. The economic size and technological progress in the emerging markets have grown considerably and they have become the drivers of the global economy (MSCI, 2023). Furthermore, the emerging markets have different country-specific characteristics and achieved different progress in gender equality as seen in Table 1.

**Table 1:** SDG-5 (Gender equality) score of emerging markets.

Country	Year	SDG 5 score	Country	Year	SDG 5 score
Brazil	2000	62.4	Kuwait	2000	49.3
	2021	69.2		2021	53.5
Chile	2000	49.7	Malaysia	2000	44.7
	2021	66.2		2021	57.3
China	2000	72.2	Mexico	2000	55.6
	2021	77.1		2021	77.9
Colombia	2000	59.7	Peru	2000	55.4
	2021	69.0		2021	65.4
Czechia	2000	66.1	Philippines	2000	51.9
	2021	72.6		2021	63.8
Egypt	2000	27.8	Poland	2000	58.3
	2021	51.0		2021	72.3
Greece	2000	45.4	South Africa	2000	71.0
	2021	64.6		2021	83.5
Hungary	2000	58.8	Thailand	2000	67.7
	2021	64.5		2021	69.4
India	2000	26.6	Turkiye	2000	29.5
	2021	33.9		2021	45.6
Indonesia	2000	55.2	United Arab Emirates	2000	40.0
	2021	62.8		2021	76.6
Korea, Rep.	2000	52.3	World	2000	50.9
	2021	65.1		2021	59.0

Sachs et al. (2022).

The empirical literature on gender inequality has generally investigated the influence of gender inequality on economic growth and development [e.g., see Kleven and Landais, 2017; Karoui and Feki, 2018; Bertay et al., 2020; Farooq et al., 2020; Koengkan et al., 2022 and Wu et al., 2022] but the empirical studies on determinants of gender inequality have stayed very limited and uncovered diverse social and economic factors behind gender inequality. In this context, Nyiransabimana (2015) and Kim (2021) focused on the influence of education on gender inequality. However, only Apergis and Lynch (2022) investigated the influence of economic freedom and education on the gender pay gap in the empirical literature. Therefore, we evaluate that this article will be one of the first studies to investigate the interplay among economic freedom, education level, and gender inequality in both the short and long term and thus targets making a contribution to the related empirical literature. The second contribution of the article is to employ cointegration and causality tests, taking notice of heterogeneity and cross-sectional properties of the panel dataset, as the empirical studies on determinants of gender inequality have usually performed a regression analysis. The subsequent section of the article scrutinizes the studies about the determinants of gender inequality, and then data and methods are disclosed. The econometric tests are performed and their results are disputed regarding the related literature in Section 4. The article arrives at a conclusion in Section 5.

## 2. Literature review

The achievement of worldwide gender equality is one of the 17 SDGs and gender equality is vital not only for the establishment of human rights but also for the achievement of other SDGs such as good health and well-being, zero hunger, no poverty, decent work, and economic growth. In this context, Nguyen (2021) analyzed the impact of gender equality on economic complexity in 119 countries for the 1991–2017 period and revealed the benefits of gender equality on economic complexity. Vyas-Doorgapersad (2019) reached the conclusion that gender equality was a significant instrument to combat poverty in South Africa. Pinho-Gomes et al. (2023) analyzed the relationship between gender equality and gender differences in life expectancy in 156 countries over the 2010–2021 period through regression analysis and revealed that higher gender equality was related to longer life expectance for women and men, but that the gender gap in life expectancy had widened. Kolip and Lange (2018) found a similar interaction between gender inequality and the gender gap in life expectancy for the European Union member states. Milner et al. (2021) also discovered that higher gender equality was associated with better health outcomes. However, the factors behind gender inequality have not been sufficiently addressed in the empirical literature yet. In this context, researchers have specified different socioeconomic factors underlying gender inequality for different countries and country groups.

Baliamoune-Lutz (2007) investigated the impact of globalization on gender inequality in Africa through regression and reached the conclusion that globalization did not have a significant impact on gender inequality in non-Sub-Saharan African developing countries, but higher global integration had a positive impact on gender inequality. Moreover, Juhn et al. (2014) developed a theoretical model of the impact of trade on gender inequality and suggested that a decrease in tariffs increases the employment of women in blue-collar jobs, but not in white-collar jobs and tested their model with data from the trade of Mexico in the context of the North American Free Trade Agreement and revealed that the findings supported their model.

Nyiransabimana (2015) researched the socioeconomic factors behind gender inequality in higher education in rural parts of Rwanda through qualitative research and uncovered that the education level of the parents, early marriage, poverty, pregnancies, and child care were the main determinants of gender inequality. Jayachandran (2015) also suggested that cultural norms were significant determinants of gender inequality in developing countries. Sangaji et al. (2018) explored the effect of GDP *per capita*, trade, and foreign direct investments on gender inequality in the Association of Southeast Asian Nations (ASEAN) members from 2010 to 2015 via dynamic regression and reached the conclusion that GDP *per capita*, foreign direct investments, and trade negatively influenced the gender inequality.

Kim (2021) examined the factors behind gender inequality in 34 countries (18 Organisation for Economic Co-operation and Development (OECD) members and 16 non-OECD states) via a regression method and reached the conclusion that compulsory education length and government educational expenditures promoted gender equality. Furthermore, fertility rate and unemployment had a positive impact on gender inequality, but all worldwide governance indicators (regulatory quality, voice and accountability, government effectiveness, rule of law, political stability, and absence of violence and corruption) and rate of employers and wage and salaried workers had a negative influence on gender inequality.

Some researchers have analyzed the interaction between education and the gender pay gap and reached different conclusions. Olarewaju et al. (2019), Adeosun and Owolabi (2021), and Akdoğan Gedik and Günel (2021) uncovered a negative influence of education on the gender pay gap, but Apergis and Lynch (2022) discovered a positive impact of education together with economic freedom on the gender pay gap.

Olarewaju et al. (2019) analyzed the influence of gender and minimum wage on returns to occupation in Nigeria with the 2004 data from the Nigerian Living Standards Survey and discovered a negative influence of education on the gender pay gap. Adeosun and Owolabi (2021) explored the determinants underlying the gender pay gap in Nigeria for the 2015–2016 period through regression and found that education decreased the gender pay gap because women had a higher return rate on education than men. Akdoğan Gedik and Günel (2021) also analyzed the determinants behind the gender wage gap in selected OECD states over the 1997–2016 period through regression analysis and they discovered that education negatively influenced the gender wage gap. However, Apergis and Lynch (2022) investigated the influence of economic freedom and education on the gender pay gap in the United Kingdom using data over the 2009–2016 period through a regression method and revealed that economic freedom and education affected the gender pay gap positively because the positive influence of economic freedom and education on wages of men were found to be higher than those of women.

Based on the related literature review, the research question of the study is:

What is the role of education level and economic freedom in gender inequality?

The hypotheses of the research are as follows:

*H1*: There is a significant relationship between education level and gender inequality.

*H2*: There is a significant relationship between economic freedom and gender inequality.

## 3. Data and method

The research investigated the influence of education level and economic freedom on gender inequality in a sample of emerging markets. Gender inequality (GII) was represented by the Gender Inequality Index of UNDP (2023). The Gender Inequality Index is calculated depending on disadvantages labor market, empowerment, and reproductive health through the association-sensitive inequality measure by Seth (2009) and gives a value between 0 (women and men fare equally) and 1 (one gender fares as poorly as possible in these three dimensions) (UNDP, 2023). On the other hand, education (EDUCATION) was proxied by the Education Index of UNDP (2023) and gives a value between 0 and 1 and higher index values show higher education levels. Last, economic freedom (EFI) was substituted by the Economic Freedom Index of the Fraser Institute (2023), and the index gives a value between 0 and 10 and higher index values indicate higher economic freedom levels. The study duration covers 2000–2020 because economic freedom data is available for this period. Furthermore, emerging markets were selected based on MSCI (2023), but Qatar, Saudi Arabia, and Taiwan were excluded from the analyses owing to data non-availability.

The major statistics of the Gender Inequality Index, Education Index, and Economic Freedom Index presented in Table 2 show that the means of the Gender Inequality Index, Education Index, and Economic Freedom Index are, respectively, 0.35, 0.6788, and 6.9152. The variation of the Economic Freedom Index among the emerging markets is relatively higher when compared those of the Gender Inequality Index and Education Index.

**Table 2:** Main characteristics of the variables.

	GII	Education	EFI
Mean	0.3500	0.6788	6.9152
Median	0.3830	0.6651	6.9300
Maximum	0.6640	0.9366	8.0400
Minimum	0.0500	0.3689	5.0000
Std. Dev.	0.1509	0.1137	0.5871
Skewness	−0.0993	0.0543	−0.2988
Kurtosis	1.8795	2.448618	2.7881

Authors own.

The interplay among economic freedom, education level, and gender inequality was analyzed by Westerlund and Edgerton (2007) using an LM (Lagrange Multiplier) bootstrap cointegration test and Dumitrescu and Hurlin (2012) causality test because a significant heterogeneity and cross-sectional dependence among three variables are unveiled. The causality analysis is useful to see the mutual interplay among economic freedom, education level, and gender inequality. Furthermore, the cointegration test examines whether the long-term linear relationship between two or more variables is stationary even if there does not exist a linear interplay in the short term (Tu et al., 2019). Therefore, the cointegration test enables us to see the long-term interplay among economic freedom, education level, and gender inequality.

The second-generation LM bootstrap cointegration test allows autocorrelation and heteroscedasticity in the cointegration equation and gives relatively more consistent results, especially for small datasets. The cointegration test based upon McCoskey and Kao’s (1998) LM test and the critical values generated from bootstrapping were considered to examine whether there was significant cross-sectional dependence among economic freedom, education level, and gender inequality (Westerlund and Edgerton, 2007). The cointegration test is generated from Equation (1):


(1) $$y_{it}=\alpha_{i}+x_{it}^{\prime }\beta_{it}+z_{it}$$

*t* = 2000,2001,….,2020 and i = 1,2….,21, respectively, show the time and cross-sections of the panel dataset and $z_{it}\left(z_{it}=\mu_{it}+v_{it}\sum_{j=1}^{t}\eta_{ij}\right)$ is the error term. $\eta_{ij}$ is an error term with a zero mean and $\sigma_{i}^{2}$ variance.

The null hypothesis of the LM bootstrap cointegration test takes a significant cointegration among economic freedom, education level, and gender inequality in the emerging markets under consideration and the existence of significant cointegration among the three variables was tested by the LM test statistic presented in Equation (2).


(2) $$LM_{N}^{+}=\frac{1}{NT^{2}}\overset{N}{\underset{i=1}{\sum }}\overset{t}{\underset{t=1}{\sum }}{\hat{\omega }}_{i}^{-2}s_{it}^{2}$$

$s_{it}^{2}$ is the partial sum of $z_{it}$ , and ${\hat{\omega }}_{i}^{-2}$ is the long-term variance of $\mu_{it}$ .The causality test analyzed the bidirectional interplay between economic freedom, education level, and gender inequality. In other words, it tests whether economic freedom has a significant influence on gender inequality and whether gender inequality has a significant influence on economic freedom. The Dumitrescu and Hurlin (2012) causality test can be performed if the panel is unbalanced or there exists heterogeneity and cross-sectional dependence, N > T, and T > N. The causality test uses the (3) numbered equation for causality analysis:


(3) $$y_{i,t}=\alpha_{i,t}\overset{K}{\underset{k=1}{\sum }}\gamma_{i}^{\left(k\right)}y_{i,t-k}+\overset{K}{\underset{k=1}{\sum }}\beta_{i}^{\left(k\right)}x_{i,t-k}+\varepsilon_{i,t}$$

In (3) numbered equation, k is lag length, γ and β are, respectively, the dependent and independent variables’ coefficients. In the causality analysis, the series under consideration should be stationary. The null hypothesis of the causality test claims a non-causality between two variables and it is tested by Wald $\left(W_{N,T}^{Hnc\;\left(Homogeneousnoncausality\right)}\right)$ and $Z_{N,T}^{Hnc}$ test statistics in equations (4) and (5):


(4) $$W_{N,T}^{Hnc}=\frac{1}{N}\overset{N}{\underset{i=1}{\sum }}W_{i,T}$$

$W_{NT}^{Hnc}$ test statistic with asymptotic distribution is considered if N<T , but the Ztild $\left(Z_{N}^{Hnc}\right)$ test statistic with a semi-asymptotic distribution is taken into account if T<N .


(5) $$Z_{NT}^{Hnc}=\sqrt{\frac{N}{2K}}\left(W_{N,T}^{Hnc}-K\right)$$

(6) $$Z_{N}^{Hnc}=\frac{\sqrt{N\left[W_{N,T}^{Hnc}-N^{-1}\sum_{i=1}^{N}E\left(W_{i,T}\right)\right]}}{\sqrt{N^{-1}\sum_{i=1}^{N}Var\left(W_{i,T}\right)}}$$

## 4. Results and discussion

The relationship between economic freedom, education level, and gender inequality was analyzed through a second-generation cointegration test and causality test. Therefore, first, cross-section dependence and heterogeneity were, respectively, examined by the tests shown in Table 3. The presence of cross-sectional dependence among economic freedom, education level, and gender inequality was tested through $LM_{adj.}$ , LM CD, and LM tests, and the tests’ findings are reported in Table 3. The alternative hypothesis of three tests (“there is significant cross-sectional dependence among three series”) was accepted because the probability values were found to be lower than 0.05. Then, the presence of homogeneity was questioned through delta tilde tests, and the tests’ results are shown in Table 3. The alternative hypothesis of the delta tests (“there is heterogeneity”) was accepted because their probability values were found to be lower than 0.05. As a result, the preference for econometric tests regarding cross-sectional dependency and heterogeneity induces us to obtain relatively more consistent results. As a result, use of unit root, cointegration, and causality tests taking notice of cross-sectional dependency and heterogeneity leads us to attain relatively more robust results.

**Table 3:** Findings of cross-sectional dependence and heterogeneity analyses.

Tests	Test statistic	*p* value
**Cross-sectional tests**		
LM _adj_ (Pesaran et al., 2008)	52.203	0.019
LM CD (Pesaran, 2004)	50.084	0.020
LM (Breusch and Pagan, 1980)	49.732	0.007
**Heterogeneity tests**		
Delta tilde (Pesaran and Yamagata, 2008)	43.509	0.000
Adjusted delta tilde (Pesaran and Yamagata, 2008)	45.176	0.000

Authors own.

The stationarity analysis of GII, EDUCATION, and EFI was performed *via* Pesaran (2007) cross-sectional augmented Dickey–Fuller (CADF) unit root test, and the findings of the unit root test are depicted in Table 4. All three series were non-stationary at level values but they became stationary at first-differenced values.

**Table 4:** Findings of CADF unit root analysis.

Variables	Level		First level	
	Constant	Constant + Trend	Constant	Constant + Trend
GII	−1.047	−1.126	−8.316***	−8.913***
EDUCATION	−0.917	−1.104	−7.587***	−8.015***
EFI	−0.874	−1.038	−6.032***	−6.811***

Authors own.

*** Significant at 1% level.

The cointegration interplay among economic freedom, education level, and gender inequality in emerging markets was analyzed by an LM bootstrap cointegration test due to cross-section dependence and a relatively small dataset. The findings of the cointegration test are reported in Table 5. As a consequence, the null hypothesis supporting the entity of significant cointegration was accepted, and in turn, it was concluded there exists a long-term relationship between economic freedom, education level, and gender inequality.

**Table 5:** Findings of LM Bootstrap cointegration analysis.

Constant			Constant + Trend		
Test statistic	Asymptotic *p* value	Bootstrap *p* value	Test statistic	Asymptotic *P* value	Bootstrap *P* value
6.324	0.218	0.317	7.202	0.342	0.419

Authors own.

Lag and lead values are 1.

Asymptotic probability values are obtained from normal distribution.

Bootstrap probability values are obtained through 10,000 simulations.

The panel and country-level cointegration coefficients were forecasted through an AMG estimator (Eberhardt and Bond, 2009; Teal and Eberhardt, 2010), and the coefficients are reported in Table 6. The panel-level coefficients uncovered that education level and economic freedom have a negative influence on gender inequality. The country-level cointegration coefficients also unveiled that education has a negative influence on gender inequality in all countries, similarly, economic freedom has a negative on gender inequality in all emerging markets except Egypt, India, the Philippines, and Kuwait. However, the negative impact of education level on gender inequality was found to be stronger than that of economic freedom in all emerging markets.

**Table 6:** Cointegration coefficient estimation.

**Countries**	**Education**	**EFI**
Brazil	−0.115***	−0.083**
Chile	−0.273**	−0.074***
China	−0.245***	−0.091***
Colombia	−0.224***	−0.103**
Czechia	−0.329***	−0.123***
Egypt	−0.183***	−0.038
Greece	−0.320***	−0.148**
Hungary	−0.317***	−0.134***
India	−0.252***	−0.175
Indonesia	−0.194**	−0.045***
Korea Republic	−0.365***	−0.139**
Kuwait	−0.331***	−0.145
Malaysia	−0.204**	−0.061**
Mexico	−0.341***	−0.118***
Peru	−0.287***	−0.089**
Philippines	−0.236***	−0.096
Poland	−0.340***	−0.141**
South Africa	−0.167***	−0.107***
Thailand	−0.265***	−0.087**
Turkiye	−0.319***	−0.105***
United Arab Emirates	−0.346***	−0.127**
Panel	−0.272***	−0.116***

Authors own.

***, and **are significant at 1 and 5% levels, respectively.

Both education level and economic freedom are expected to decrease gender inequality depending on country-specific characteristics through raising the awareness of society about the role of women in economic and social development and introducing equal opportunities for everybody regardless of gender. However, human development, cultural norms, and institutional and legal factors in the countries are significant for the interplay among education level, economic freedom, and gender inequality (Jayachandran, 2015; Kim, 2021). In the empirical literature, Nyiransabimana (2015) and Kim (2021) focused on the factors underlying gender inequality in Rwanda and OECD countries, respectively. Nyiransabimana (2015) suggested the education level of parents together with other socioeconomic factors were significant determinants of gender inequality. Kim (2021) uncovered that compulsory education length and government educational expenditures were determinants of gender equality with public governance indicators and other socioeconomic factors. Furthermore, Jayachandran (2015) suggested that cultural norms were significant determinants of gender inequality in developing countries and Sangaji et al. (2018) also found that GDP *per capita*, foreign direct investments, and trade were significant determinants of gender inequality. Therefore, the limited empirical literature supports the significant role of education in gender inequality uncovered by the cointegration analysis in this study and also suggests that country-specific characteristics such as economic development, cultural norms, and public governance influence the interaction between education level and gender inequality.

On the other hand, some researchers have analyzed the impact of education and economic freedom on the gender pay gap and reached different results. Olarewaju et al. (2019), Adeosun and Owolabi (2021), and Akdoğan Gedik and Günel (2021) uncovered a negative influence of education on the gender pay gap, but Apergis and Lynch (2022) discovered a positive impact of education and economic freedom on the wages of women and men, but the positive impact of education and economic freedom on the wages of men was found to be relatively higher than those of women. So, these studies also verify that education and economic freedom have a positive influence on gender equality by decreasing the gender pay gap.

Furthermore, a negative influence of education level on gender inequality is discovered for all emerging markets under consideration, but the size of the cointegration coefficients remarkably varies among emerging markets. The findings uncover that countries with higher human and economic development such as Czechia, Greece, Hungary, the Republic of Korea, Kuwait, Malaysia, Poland, Thailand, Turkiye, and the United Arab Emirates generally experience a higher negative impact of education level and economic freedom on gender inequality.

The causal relationship between education level, economic freedom, and gender inequality was analyzed by the Dumitrescu and Hurlin (2012) causality test, and the findings of the causality analysis are reported in Table 7. The findings of causality analysis support the theoretical expectations of a two-way causal relationship between education level, economic freedom, and gender inequality because both education level and economic freedom have a significant influence on gender inequality and gender inequality also has a significant impact on education level and economic freedom by raising the role of women in social and economic life. In the empirical literature, the two-way interplay between economic freedom, education level, and gender inequality has not been analyzed yet. Therefore, this study will be useful to see the mutual interaction among these variables.

**Table 7:** Findings of causality test.

**Null hypothesis**	**Test**	**Test statistics**	***P* value**
DEDUCATION ↛DGII	*Whnc*	6.204	0.000
	*Zhnc*	6.873	0.000
	*Ztild*	7.214	0.000
DGII ↛DEDUCATION	*Whnc*	8.462	0.000
	*Zhnc*	8.977	0.000
	*Ztild*	9.104	0.003
DEFI ↛DGII	*Whnc*	8.255	0.000
	*Zhnc*	8.607	0.000
	*Ztild*	9.056	0.000
DGII ↛DEFI	*Whnc*	6.352	0.000
	*Zhnc*	6.911	0.000
	*Ztild*	7.283	0.000

Authors own.

## 5. Conclusion

The world has already had significant gender gaps in all aspects of life such as economic and political participation, education, and health. Therefore, gender equality is accepted as one of the 17 SDGs. Furthermore, gender equality is also a critical factor for the achievement of other SDGs considering that women constitute half of the human capital in the world. However, nearly 16% improvement has been achieved in gender equality (SDG-5) between 2000 and 2021. Therefore, more struggles and measures to reach gender equality globally are required. Similarly, we reveal that studies on the factors underlying gender inequality have been limited. The limited literature motivated us to analyze the interplay among economic freedom, education level, and gender inequality in a sample of emerging markets. Data availability limited our study period and sample. The study period was specified as 2000–2020, because annual economic freedom data was available over the 2000–2020 duration, and the emerging markets of Qatar, Saudi Arabia, and Taiwan were not included in the econometric analyses owing to data absence.

The findings of causality analysis uncovered a two-way interplay between economic freedom, education level, and gender inequality in keeping with theoretical considerations. In other words, gender inequality, economic freedom, and education level impact each other in the short term. On the other hand, the findings of the cointegration analysis indicated that economic freedom and education level have a negative impact on gender inequality in the long run, but the negative impact of education level on gender inequality was unveiled to be relatively stronger than that of economic freedom in all emerging markets. Furthermore, the findings of the long-term analysis showed that the negative impact of education level and economic freedom on gender inequality is higher in countries with higher human and economic development and institutional quality. As a consequence, both education and market-oriented economic structures are significant instruments to decrease gender inequality in both the short and long term. However, country-specific characteristics such as economic development, public governance, cultural norms, customs, and traditions have an influence on the impact of education and economic freedom on gender inequality. Therefore, countries should consider their country-specific characteristics when designing educational and market-oriented policies to improve gender equality. Future studies can focus on the role that cultural and institutional norms have in the interplay among education level, economic freedom, and gender inequality.

## Data availability statement

The raw data supporting the conclusions of this article will be made available by the authors, without undue reservation.

## Ethics statement

Ethical review and approval was not required for the study on human participants in accordance with the local legislation and institutional requirements. Written informed consent from the participants was not required to participate in this study in accordance with the national legislation and the institutional requirements.

## Author contributions

Conceptualization: PT, GS, MD and MAT. Formal analysis: PT, GS, MD and MAT. Investigation: PT, GS, MD and MAT. Methodology: PT, GS, MD and MAT. Supervision: PT and GS. Writing – original draft and editing: PT, GS, MD and MAT. All authors have read and agreed to the published version of the manuscript.

## Conflict of interest

The authors declare that the research was conducted in the absence of any commercial or financial relationships that could be construed as a potential conflict of interest.

## Publisher’s note

All claims expressed in this article are solely those of the authors and do not necessarily represent those of their affiliated organizations, or those of the publisher, the editors and the reviewers. Any product that may be evaluated in this article, or claim that may be made by its manufacturer, is not guaranteed or endorsed by the publisher.
